# Increased prevalence of anxiety and depression symptoms in patients with coronary artery disease before and after percutaneous coronary intervention treatment

**DOI:** 10.1186/s12888-016-0972-9

**Published:** 2016-07-22

**Authors:** Guoqiang Gu, Yaqing Zhou, Ying Zhang, Wei Cui

**Affiliations:** Department of Cardiology, The Second Hospital of Hebei Medical University, No. 215, He Ping Xi Road, Shijiazhuang, 05000 China

**Keywords:** Anxiety and depression symptoms, Coronary artery disease, Percutaneous coronary intervention

## Abstract

**Background:**

Evidence suggests that coronary heart disease (CHD) is associated with increased anxiety and a high incidence of comorbid anxiety and depression. However, the association between percutaneous coronary intervention (PCI) and comorbid anxiety and depression has not been previously investigated. This study aims to determine the relationship between PCI and anxiety and depression symptoms in CHD patients in terms of the occurrence, prone factors, and long-term outcomes of CHD.

**Methods:**

One hundred seventy CHD patients who underwent PCI treatment between September 2013 and February 2014 at the Second Hospital of Hebei Medical University were randomly selected. All patients independently completed the Hospital Anxiety and Depression Scale (HADS) and a preoperative questionnaire; they also provided details regarding their PCI-related concerns one day before PCI, as well as one day and one, three, six, and 12 months after PCI.

**Results:**

PCI treatment was significantly associated with the symptoms of anxiety, depression, affective disorders, and comorbid anxiety and depression (χ^2^ = 90.18, 54.45, 101.59, 64.83; *p* < 0.01) at each follow-up time point. Moreover, PCI treatment was linearly correlated with each of these psychological issues (*p* < 0.01).

**Conclusion:**

The prevalence of anxiety, depression, and comorbid anxiety and depression symptoms significantly increases one day before and after PCI treatment; however, the incidence of these psychological issues significantly decreases with time following PCI. A low level of education, apprehension with regard to nursing quality, potential cardiac dysfunction, surgery sequelae, and surgery failure are also associated with a high prevalence of anxiety and depression symptoms.

## Background

Anxiety and depression symptoms are commonly associated with coronary heart disease (CHD). A recent systematic review indicated that the overall prevalence of depression in patients with CHD in China was 51 % [1], among which 3.1–11.2 % of individuals suffered from severe depression [1]. Previous studies have suggested that depression is a risk factor for poor prognosis of CHD [2–4]. Other evidence has also indicated that depression increases the risk of myocardial infarction in CHD patients [5, 6]. CHD patients with depression are particularly vulnerable to myocardial reperfusion injury when they face psychological challenges, thus increasing the recurrence of acute coronary syndrome and mortality [5, 6]. Another psychological factor negativly associated with cardiovascular disease is anxiety, which may occur alone or in combination with depression. Watkins demonstrated that the simultaneous occurrence of anxiety and depression had a significant adverse impact on the prognosis of CHD [2]. Thus, the importance of focusing on comorbid anxiety and depression in CHD patients has been emphasized [3]. Importantly, these findings indicate that anxiety and depression are both risk factors for CHD and play important roles in the initiation, development, rehabilitation, and prognosis of CHD.

Currently, percutaneous coronary intervention (PCI) is considered one of the primary approaches for CHD treatment. PCI significantly alleviates the symptoms of CHD, reduces mortality, and substantially improves quality of life [7]. The number of patients who receive PCI treatment has increased annually. However, some patients may develop psychological issues after PCI treatment because of several factors [8–10], including a lack of 1) knowledge regarding the disease and surgical intervention; 2) sufficient medical education; or 3) correct information on the surgical procedure. These psychological issues may significantly affect a patient’s daily life and work activities. Previous studies have demonstrated that in general, patients who underwent PCI surgery developed affective disorders, including anxiety and depression [11, 12]. However, the associations between PCI and anxiety and depression symptoms in CHD patients in terms of occurrence, risk factors, and the long-term outcomes of CHD remain unknown. To the best of our knowledge, this is the first study that aims to investigate the incidence of anxiety and depression symptoms, risk factors, and long-term outcomes of CHD patients undergoing PCI.

## Methods

### Subjects

One hundred seventy hospitalized CHD patients (120 males and 50 females, aged 33–80 years old), who underwent PCI at the Department of Internal Cardiology in our hospital between September 2013 and February 2014, were enrolled in this study. This study was approved by the Ethics Committee of the Second Hospital of Hebei Medical University. All patients were enrolled after providing written informed consent.

The inclusion criteria were as follows: age ≥ 18 years; PCI was required based on coronary angiography results, individuals voluntarily participated in the psychological evaluation.

Exclusion criteria included a history of at least one of the following conditions: mental illness or history of prophylactics for mental illness; valvular heart disease and/or cardiomyopathy; cognitive impairment; stroke; or nervous system disorder due to liver, lung, kidney, or other diseases. Individuals were also excluded because of a lack of ability to complete the psychological evaluation due to severe illness, surgical emergency, or lack of cooperation.

### Methods

#### Assessment tools and methods

Hospital Anxiety and Depression Scale (HADS) consists of 14 items, including 7 items that assess anxiety symptoms and 7 items that assess depression symptoms. The scale was divided into two factors based on the CCMD-2 (Chinese Classification of Mental Disorders-2) diagnostic criteria, and the Self-Rating Depression Scale (SDS) and Self-Rating Anxiety Scale (SAS) were used as a reference for comparative study. The threshold for anxiety or depression was 8 points [13].

Preoperative and postoperative psychological questionnaires were administered.

1. The preoperative questionnaire assessed the following variables: 1) attitude towards CHD; 2) attitude towards PCI treatment of CHD; 3) preoperative mood; 4) views regarding prospects for stent treatment; 5) requirements regarding surgeon; and 6) most significant concern prior to surgery.
2. The postoperative questionnaire assessed the following items: 1) knowledge of the surgeon who conducted the operation; 2) degree of satisfaction regarding the stent therapy; 3) mood after PCI treatment; 4) reconsideration of PCI; 5) physical symptoms after PCI; and 6) the most significant concern after surgery.

A three-stage system was applied to evaluate the first five questions in the questionnaire. For example, the requirement for the surgeon was divided into three levels, including not strict, general, and very strict, represented by scores of 1, 2, and 3 points, respectively. The sixth question consisted of 12 and 11 selectable items, such as a fear of impaired heart function, sequela after surgery, failure to receive attentive care, surgery failure, and recurrence of CHD. Responses were divided into two levels, concern and no-concern.

#### Main testing indicators and measurement methods

1. The indicators related to anxiety and depression symptoms include anxiety and depression scores on the HADS, preoperative questionnaire, preoperative concerns, understanding of the disease prior to surgery, knowledge of the surgeon, understanding of the surgery, postoperative questionnaire, and postoperative concerns.
2. Surgery-related issues include the diagnosis of angina or myocardial infarction, diseased left main artery, number of diseased blood vessels, blood vessel where stent was placed, whether blood vessel was completely processed, amount of heparin used in operation, and amount of contrast agent used in operation.
3. Disease history refers to the disease duration and a history of hypertension, diabetes, hyperlipidemia, or cerebral vascular disease.
4. The general demographic information includes age, sex, work location, education, marriage, and children. The education levels were divided into five categories, including illiteracy, elementary or junior high school, high school or secondary vocational school, university or junior college, and graduate and above.
5. Supplementary examination indicators include blood test results, high-sensitivity C-reactive protein level, and liver and kidney function.
6. Assessment of major adverse cardiovascular events (MACE). The follow-up endpoint comprised the occurrence of MACE within a 12-month follow-up period, which included non-fatal myocardial infarction, target lesion revascularization (TLR), and all-cause death. Acute myocardial infarction refers to an acute ST-segment elevation and an acute non-elevation myocardial infarction. TLR refers to the presence of vascular disease within 5 mm at bilateral ends of the stent, which requires renewed PCI or coronary artery bypass graft surgery. The mode of death includes cardiac and non-cardiac death.

### Procedures

All procedures were supervised by trained cardiologists, who tested each patient one day before and after surgery. All patients were allowed to independently complete the questionnaires within 30 min. Patients who had difficulties reading and/or understanding the questions presented in the questionnaires were provided with limited counseling to complete the assessments. Follow-up assessments were performed either in the outpatient department or *via* telephone at 1, 3, 6, and 12 months after surgery, at which point anxiety and depression were assessed. To assess anxiety symptoms, the scores were divided into two categories: the anxious symptoms group (HADS-A ≥ 8 points) and the non-anxious symptoms group (HADS-A < 8 points). To assess depression, the scores were divided into two categories: the depressive symptoms group (HADS-D ≥ 8 points) and the non-depressive symptoms group (HADS-D < 8 points). The levels of anxiety and depression symptoms and the presence of MACE were evaluated.

### Statistical analysis

1. SPSS version 13.0 (SPSS, Chicago, IL, USA) was used for statistical analysis.
2. Data are presented as the mean ± standard deviation (SD) for measurement data; the count data are expressed as percentages. Each set of data was tested regarding normality and homogeneity of variance. If data were normally distributed, independent sample *t*-tests were used for comparisons between two data sets. Otherwise, a non-parametric test was used for data analysis. The population rate or the constituent ratio of the count data were analyzed using Chi-square tests.
3. The prevalence of anxiety and depression symptoms at different time points was compared using Chi-square tests. A linear trend test and post-multiple comparison were used to analyze the relationship between the prevalence of anxiety and depression symptoms and PCI at each time point.
4. A multivariate logistic regression analysis was used to analyze factors related to disease prevalence. Demographic characteristics (such as gender and age) and clinical characteristics (such as type of disease, family history, high blood pressure, and diabetes) were adjusted in the multivariate analysis process. *P* < 0.05 was considered statistically significant.

## Results

### General characteristics

Of the 170 patients, 59 (34.70 %) patients, with a mean age of 58 years (range: 50–64 years), had symptoms of anxiety on the day before surgery, and 68.49 % of these cases were male. Forty (23.50 %) patients with a mean age of 58.50 years (range: 50.25–64.75 years) had symptoms of depression (60.00 % male; Table 1).

**Table 1:** Prevalence of anxiety and depression based on demographic and clinical indicators one day before PCI

Parameters	Group			Group		
n (%)	Anxiety	Non-anxiety	*P*	Depression	Non-depression	*P*
	(*n* = 59)	(*n* = 111)		(*n* = 40)	(*n* = 130)	
Female	18 (36.00)	32 (64.00)	0.819	16 (32.00)	34 (68.00)	0.093
Alone	3 (21.40)	11 (78.60)	0.384	7 (50.00)	7 (50.00)	0.023
Occupation						
Farmers	35 (38.00)	57 (62.00)	0.591	29 (31.50)	63 (68.50)	0.014
Workers	11 (32.40)	23 (67.60)		7 (20.60)	27 (79.40)	
Services	13 (29.50)	31 (70.50)		4 (9.10)	40 (90.90)	
Reimbursement						
Expense	5 (45.50)	6 (54.50)	0.043*	2 (18.20)	9 (81.80)	0.014
Provincial health insurance	8 (20.50)	31 (79.50)		6 (15.40)	33 (84.60)	
NCMS	39 (42.90)	52 (57.10)		30 (33.00)	61 (67.00)	
Medicare places	7 (24.10)	22 (75.90)		2 (6.90)	27 (93.10)	
Monthly income						
<1000 yuan	27 (39.10)	42 (60.90)	0.373	26 (37.70)	43 (62.30)	0.001
1000–3000 yuan	27 (30.00)	63 (70.00)		11 (12.20)	79 (87.80)	
3000–5000 yuan	5 (50.00)	5 (50.00)		3 (30.00)	7 (70.00)	
>5000 yuan	0 (0.00)	1 (100.00)		0 (0.00)	1 (100.00)	
Work location						
City	9 (23.10)	30 (76.90)	0.142	4 (10.30)	35 (89.70)	0.039
County	14 (32.60)	29 (67.40)		9 (20.90)	34 (79.10)	
Rural	36 (40.90)	52 (59.10)		27 (30.70)	61 (69.30)	
Education						
Illiteracy	9 (75.00)	3 (25.00)	0.042	8 (66.70)	4 (33.30)	0.004
Primary or junior high school	32 (33.00)	65 (67.00)		24 (24.70)	73 (75.30)	
High school or secondary vocational school	15 (30.60)	34 (69.40)		7 (14.30)	42 (85.70)	
Bachelor’s degree or junior college	3 (27.30)	8 (72.70)		1 (9.10)	10 (90.90)	
Master’s degree or above	0 (0.00)	1 (100.00)		0 (0.00)	1 (100.00)	

On the day before surgery, 74 (43.50 %) patients with a mean age of 58 years (range: 50.00–64.00) had symptoms of either anxiety or depression (67.57 % male). Furthermore, 25 (14.70 %) patients with a mean age 58 years (range: 50.00–64.00 years) had symptoms of comorbid anxiety and depression (60.00 % male; Table 2).

**Table 2:** Prevalence of comorbid anxiety and depression based on different demographic and clinical indicators one day before PCI

Parameters	Affective disorder (*n* = 74)	Control group (*n* = 96)	*P*	Comorbid group (*n* = 25)	Non-comorbid group (*n* = 145)	*P*
n (%)						
Female	24 (48.00)	26 (52.00)	0.448	10 (20.00)	40 (80.00)	0.208
Alone	7 (50.00)	7 (50.00)	0.610	3 (21.40)	11 (78.60)	0.437
Occupation						
Farmers	46 (50.00)	46 (50.00)	0.129	18 (19.60)	74 (80.40)	0.126
Workers	14 (41.20)	20 (58.80)		4 (11.80)	30 (88.20)	
Services	14 (31.80)	30 (68.20)		3 (6.80)	41 (93.20)	
Reimbursement						
Expense	6 (54.50)	5 (45.50)	0.004	1 (9.10)	10 (90.90)	0.089
Provincial health insurance	10 (25.60)	29 (74.40)		4 (10.30)	35 (89.70)	
NCMS	50 (54.90)	41 (45.10)		19 (20.90)	72 (79.10)	
Medicare places	8 (27.60)	21 (72.40)		1 (3.40)	28 (96.60)	
Monthly income						
<1000 yuan	38 (55.10)	31 (44.90)	0.031	15 (21.70)	54 (78.30)	0.025
1000–3000 yuan	31 (34.40)	59 (65.60)		7 (7.80)	83 (92.20)	
3000–5000 yuan	5 (50.00)	5 (50.00)		3 (30.00)	7 (70.00)	
>5000 yuan	0 (0.00)	1 (100.00)		0 (0.00)	1 (100.00)	
Work location						
City	9 (23.10)	30 (76.90)	0.009	4 (10.30)	35 (89.70)	0.211
County	19 (44.20)	24 (55.80)		4 (9.30)	39 (90.70)	
Rural	46 (52.30)	42 (47.70)		17 (19.30)	71 (80.70)	
Education						
Illiteracy	10 (83.30)	2 (16.70)	0.010	7 (58.30)	5 (41.70)	0.003
Primary or junior high school	45 (46.40)	52 (53.60)		11 (11.30)	86 (88.70)	
High school or secondary vocational school	16 (32.70)	33 (67.30)		6 (12.20)	43 (87.80)	
Bachelor’s degree or junior college	3 (27.30)	8 (72.70)		1 (9.10)	10 (90.90)	
Master’s degree or above	0 (0.00)	1 (100.00)		0 (0.00)	1 (100.00)	

### Determination of factors impacting anxiety and depression symptoms at different time points

The symptoms of anxiety, depression, affective disorders, and comorbid anxiety and depression comprised dependent variables. A univariate analysis of risk factors was conducted with age, sex, family history of depression or anxiety as the independent variables. A multivariate logistic regression analysis was performed. Following adjustment for demographic and clinical characteristics, as well as other confounding factors, a high level of education was identified as a protective factor against preoperative symptoms of anxiety, depression, affective disorders, and comorbid anxiety and depression. Odds ratios (ORs) were 0.56 (95 % confidence interval [CI], 0.34–0.91), 0.34 (95 % CI, 0.19–0.66), 0.44 (95 % CI, 0.26–0.73), and 0.430 (95 % CI, 0.21–0.88), respectively. The fear of surgery sequelae was a risk factor for preoperative symptoms of anxiety with an OR of 2.46 (95 % CI, 0.34–0.91). Concerns regarding the quality of nursing and potential cardiac function impairment were risk factors for preoperative symptoms of depression with ORs of 4.03 (95 % CI, 1.14–12.33) and 3.11 (95 % CI, 1.37–7.06), respectively. Fear of nursing quality and cardiac function impairment were risk factors for affective disorders symptoms of with ORs of 3.61 (95 % CI, 1.20-10.87) and 2.09 (95 % CI, 1.02-4.27), respectively. Furthermore, fear of heart function impairment was a risk factor for symptoms of comorbid anxiety and depression with an OR of 3.72 (95 % CI, 1.52-9.13; Table 3).

**Table 3:** Binary logistic regression analysis for factors related to the prevalence of anxiety and/or depression in different periods of PCI

	Symptom classification	Parameters	OR	95 % CI	*p* value
1 day before operation	Anxiety	High education level	0.56	0.34–0.91	0.02*
		Surgical complications	2.46	1.23–4.94	0.011*
	Depression	High education level	0.34	0.18–0.66	0.001**
		*Inattentive Care*	4.03	1.14–12.33	0.014*
		Cardiac dysfunction	3.11	1.37–7.06	0.007**
	Affective disorder	High education level	0.44	0.27–0.73	0.001**
		*Inattentive Care*	3.61	1.20–10.87	0.023*
		Surgical complications	2.09	1.03–4.27	0.044*
	Cormorbid anxiety and depression	High education level	0.43	0.21–0.88	0.02*
		Cardiac dysfunction	3.72	1.52–9.13	0.004**
1 day after operation	Anxiety	Cardiac dysfunction	2.67	1.12–6.41	0.028*
	Comorbid anxiety and depression	Cardiac dysfunction	2.38	1.05–5.41	0.038*
12 months after operation	Anxiety	Symptoms present	3.14	1.31–7.51	0.01*
	Depression	Symptoms present	3.02	1.32–6.96	0.009**
		History of drinking	0.25	0.07–0.89	0.032*
	Affective disorder	Symptoms present	2.79	1.27–6.14	0.011*
	Comorbid anxiety and depression	Symptoms present	4.74	1.81–12.38	0.002**

*P < 0.05; **P < 0.01; *OR* odds ratio, *CI* confidence interval

Concern over cardiac function damage was a risk factor for postoperative symptoms of anxiety and comorbid anxiety and depression with ORs of 2.67 (95 % CI, 1.12-6.41) and 2.38 (95 % CI, 1.05-5.41), respectively (Table 3).

Next, the symptoms of anxiety, depression, affective disorders, and comorbid anxiety and depression were considered dependent variables, and smoking, drinking, amount of physical activity, regular medication, and family history of affective disorders during 12-month follow-up were considered independent variables. During the 12 months after surgery, presence of symptoms was a risk factor for anxiety, depression, affective disorders, and comorbid anxiety and depression, with ORs of 3.14 (95 % CI, 1.31–7.51), 3.03 (95 % CI, 1.32–6.96), 2.79 (95 % CI, 1.27–6.14), and 4.74 (95 % CI, 1.81–12.38), respectively. Unexpectedly, a history of drinking was a protective factor against depression symptoms, with an OR of 0.254 (95 % CI, 0.07–0.89; Table 3).

### Changes in the prevalence of anxiety and depression over time

**Fig. 1:**
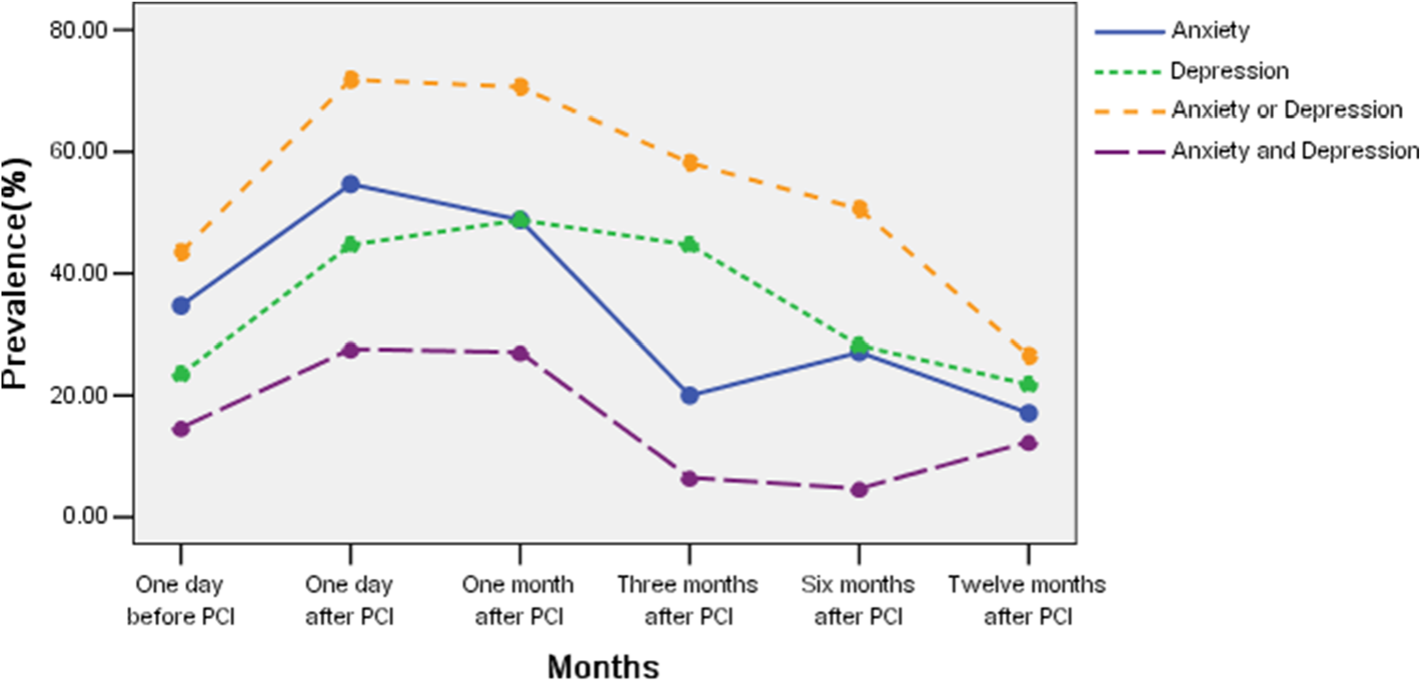
Change trend in prevalence of anxiety and/or depression during different periods of percutaneous coronary intervention

The prevalence of anxiety symptoms at each follow-up time point was significantly different (χ^2^ = 90.18; *p* < 0.01), and it linearly correlated with the time trend (*p* < 0.01). One day before surgery, the occurrence rate of anxiety symptoms was 34.70 % (59/170), which significantly increased one day after surgery (54.70 %, χ^2^ = 13.75; *p* < 0.001). There was no significant difference in the prevalence of anxiety symptoms between one day and 1 month after surgery (χ^2^ = 1.18; *p* = 0.278); however, the prevalence was significantly decreased three months after the operation (χ^2^ = 31.29; *p* < 0.001). There was no significant difference in the prevalence of anxiety symptoms between the 3rd and 6th or 12th months (χ^2^ = 2.17, *p* = 0.141 and χ^2^ = 0.49, *p* = 0.485, respectively; Fig. 1, Table 4).

**Table 4:** Changes in the trends of anxiety and/or depression prevalence at different PCI time points

Time	Depression (%)	*p*	Afterwards, multiple comparisons	Anxiety (%)	*p*	Afterwards, multiple comparisons
1 day before surgery (T_e_)	40 (23.50)	0.00**	T_e_ < T_0_*** T_e_ < T_1_*** T_e_ < T_3_*** T_0_ > T_6_*** T_0_ > T_12_*** T_1_ > T_6_*** T_1_ > T_12_*** T_3_ > T_6_*** T_3_ > T_12_***	59 (34.70)	0.00**	T_e_ < T_0_*** T_e_ > T_3_*** T_e_ > T_12_*** T_0_ > T_3_*** T_0_ > T_6_*** T_0_ > T_12_*** T_1_ > T_3_*** T_1_ > T_6_*** T_1_ > T_12_***
1 day after surgery (T_0_)	76 (44.70)			93 (54.70)		
1 month (T_1_)	83 (48.80)			83 (48.80)		
3 months (T_3_)	76 (44.70)			34 (20.20)		
6 months (T_6_)	48 (28.20)			46 (26.70)		
12 months (T_12_)	37 (21.80)			29 (17.10)		

**P < 0.01;***P < 0.003

Similar to the findings related to anxiety symptoms, a significant difference (χ^2^ = 54.45; *p* < 0.01) in the prevalence of depression symptoms after PCI was identified at each time point, which linearly correlated with the time change trend (*p* < 0.01). One day before surgery, the prevalence of depression symptoms was 23.50 % (40/170), and it was significantly increased one day after surgery (χ^2^ = 16.96; *p* < 0.001). There was no significant difference in the occurrence rate of depression symptoms between the 1st day postoperation and the 1st or 3rd months (χ^2^ = 0.58, *p* = 0.447 and χ^2^ = 0.00, *p* = 1.000, respectively). However, the prevalence of depression at the 6th month time point was significantly decreased compared with the 3rd month (χ^2^ = 9.95; *p* < 0.003); however, no significant difference was identified compared with the 12th month (χ^2^ = 1.90, *p* = 0.168; Fig. 1, Table 4).

There was a significant difference (χ^2^ = 101.59; *p* < 0.01) in the prevalence of affective disorders symptoms after PCI at each follow-up time point, which changed linearly with time (*p* < 0.01). A total of 43.50 % (74/170) of the patients exhibited an affective disorder one day before PCI. One day after surgery, the percentage of patients who had symptoms of an affective disorder was significantly increased (χ^2^ = 27.76; *p* < 0.001). There was no significant difference in the occurrence of symptoms of mood disorders between the 1st and 3rd month time points (χ^2^ = 0.06, *p* = 0.811 and χ^2^ = 6.84, *p* = 0.009, respectively); however, the prevalence was significantly lower at the 6th month (χ^2^ = 14.24; *p* < 0.001) and further decreased at the 12th month follow-up time point (χ^2^ = 20.88, *p* < 0.001; Fig. 1, Table 5).

**Table 5:** Change trend of anxiety and/or depression prevalence during different periods of PCI

Time	Depression	*P*	Afterwards multiple comparisons	Anxiety	*P*	Afterwards multiple comparisons
	n (%)			n (%)		
1 day before surgery (T_e_)	74 (43.50)	0.00**	T_e_ < T_0_*** T_e_ < T_1_*** T_6_ < T_e_*** T_12_ < T_e_*** T_0_ > T_6_*** T_0_ > T_12_*** T_1_ > T_6_*** T_1_ > T_12_*** T_3_ > T_12_*** T_6_ > T_12_***	25 (14.70)	0.00**	T_e_ < T_0_*** T_e_ > T_3_*** T_e_ > T_6_*** T_e_ > T_12_*** T_0_ > T_6_*** T_1_ > T_3_*** T_1_ > T_6_*** T_1_ > T_12_***
1 day after surgery (T_0_)	122 (71.80)			47 (27.60)		
1 month (T_1_)	120 (70.60)			46 (27.10)		
3 months (T_3_)	99 (58.20)			11 (6.50)		
6 months (T_6_)	86 (50.60)			8 (4.70)		
12 months (T_12_)	45 (26.50)			21 (12.40)		

**P < 0.01; ***P < 0.003

The prevalence of comorbid anxiety and depression symptoms was significantly different and linearly correlated with time (χ^2^ = 64.83; *p* < 0.01). One day after surgery, the number of patients with comorbid anxiety and depression symptoms was significantly increased compared with one day before surgery (χ^2^ = 8.53; *p* < 0.0033); however, there was no significant difference between one day and 1 month after surgery (χ^2^ = 0.02; *p* = 0.903), and the comorbidity rate was significantly lower at the 3rd month compared with the 1st month (χ^2^ = 25.82; *p* < 0.001). After 3 months, there were no significant changes during the 12-month follow-up period in comorbid symptoms (χ^2^ = 0.50, *p* = 0.479 and χ^2^ = 3.45, *p* = 0.063 for 3 and 12 months, respectively; Fig. 1, Table 5).

### MACE

No MACE were identified during the 12-month follow-up period.

## Discussion

In the present study, we investigated the prevalence of anxiety symptoms, depression symptoms, and comorbid anxiety and depression symptoms in CHD patients after PCI. Our findings indicated that the prevalence of these symptoms significantly increased one day before and after PCI treatment; however, these rates significantly decreased over time after PCI. In addition, a high level of education protected patients from developing preoperative symptoms of anxiety, depression, and comorbid anxiety and depression. Patients who exhibited apprehension with regard to nursing quality, potential cardiac dysfunction, surgery sequelae, and surgery failure also exhibited a high prevalence of anxiety and depression symptoms.

Prior to PCI, the prevalence rates of anxiety symptoms, depression symptoms, affective disorders symptoms, and comorbid anxiety and depression symptoms were substantially increased compared with the general population. Previous studies [2, 3, 14] have also demonstrated that CHD patients had increased anxiety symptoms and a high incidence of comorbid anxiety and depression symptoms. Simmonds reported that the prevalence of depression symptoms in elderly patients with CHD was 60.7 % and was associated with family function [15]. Frasurc-Smith studied 785 hospitalized patients with acute myocardial infarction and determined that 69 % of the patients developed various types of anxiety symptoms [14]. Our study indicated that the reason why patients exhibited increased levels of anxiety and depression before PCI may be attributed, at least in part, to the patients’ concerns regarding nursing quality, potential heart function damage, surgical sequelae, and surgery failure. Furthermore, other reasons that accounted for the increased prevalence of psychological issues in CHD patients after PCI may include anxiety induced by the frequent onset of CHD symptoms and the operation itself.

Our study also identified a significantly increased prevalence of anxiety symptoms, depression symptoms, and comorbid anxiety and depression symptoms after PCI. The patients with comorbid anxiety and depression obtained a lower score on the postoperative questionnaire following surgery, which suggested that these patients had limited understanding of the disease and surgery, exhibited lower satisfaction with the surgery, and felt physically unwell following surgery. In addition, a fear of the risks associated with PCI treatment and the potential complications, such as in-stent restenosis and stent thrombosis, were likely to potentiate mental stress, which may contribute to the postoperative incidence rates of anxiety and depression symptoms. The exact mechanisms that underlie the increased occurrence rate of comorbid anxiety and depression symptoms after PCI are not clear. However, some studies suggest that chronic anxiety is typically accompanied by a series of pathophysiological processes, including increased sympathetic nervous system activity [13] and inflammation [16–19]. Moreover, anxiety or depression symptoms has been demonstrated to increase the inflammatory response in CHD patients [20]. Additionally, the surgery itself may increase sympathetic activity and inflammatory reactions.

Our current findings also demonstrated that changes in anxiety and depression symptoms over time after PCI exhibited a linear correlation with time. Following PCI, the prevalence of postoperative anxiety, comorbid anxiety, and depression symptoms increased for approximately one month, whereas the prevalence of depression and mood disorder symptoms persistently increased for approximately three months. These results are in contrast with a previous report [21], in which acute myocardial infarction (AMI) patients were followed for 18 months and exhibited increased anxiety, but not depression, at the early stage of AMI. After 3–18 months, the levels of anxiety and depression were similar to the control population. The discrepancies between the previous and current studies may have resulted from the differences in the patient characteristics and the durations of follow-up study. In the present study, the changes in the prevalence of anxiety and depression symptoms were most likely due to the following reasons: I) the blood flow to the ischemic areas was gradually restored following PCI, which subsequently reduced the occurrence of anxious and depressive symptoms caused by ischemia; II) potential complications, such as heart function damage, which lead to concerns, did not occur with time after PCI: thus, the patients’ anxiety and depression symptoms may have been alleviated; and III) following the improvement in symptoms, the patients gradually resumed normal daily activities and aerobic exercise, which has been demonstrated to alleviate the symptoms of depression in CHD patients similar to the outcomes of psychotherapy and antidepressant treatment [22]. Exercise therapy is also currently considered a core component of cardiac rehabilitation [23], which has been proven to effectively reduce the incidence of depression symptoms and cardiac events, improve patients quality of life, and subsequently diminish the risk of CHD morbidity and mortality [22, 24, 25].

A high level of education was also demonstrated to protect patients from developing preoperative symptoms of anxiety, depression, and comorbid anxiety and depression. This protection was most likely attributed to the fact that the patients with a lower educational level had limited knowledge regarding their illness and exhibited increased concern regarding the adverse consequences of the disease. Furthermore, concern over potential cardiac function damage on day 1 following the operation was a risk factor for postoperative symptoms of anxiety alone, as well as comorbid anxiety and depression. Taken together, patients with a low education level or fear of nursing quality, potential cardiac dysfunction, surgery sequelae, or surgery failure exhibited an increased prevalence of anxiety and depression symptoms.

In the present study, the patients did not exhibit MACEs during the 12-month follow-up, which may be because the patients enrolled in this study were scheduled for PCI treatment, and the surgery was well prepared ahead of time. However, we could not rule out the possibility that the lack of MACE may be associated with the small sample size and short follow-up period. The current finding is different from several previous studies demonstrating that depression increased short-term mortality in CHD patients [23, 26–28]. Several studies have also indicated that the long-term prognosis of CHD patients was significantly correlated with depression [29–31]. However, not all studies have identified a link between anxiety and adverse clinical outcomes in CHD patients; for example, no apparent correlation was identified between anxiety and the mortality rate of hospitalized patients with AMI [32–34]. Therefore, additional investigations with a larger sample size and longer follow-up time period are required to clarify the impact of anxiety and depression symptoms on the prognosis of patients after PCI.

However, there are several study limitations that must be considered in the interpretation of our findings. First, the HADS is widely used by physicians in general hospitals to assess suspected anxiety or depression disorders in patients. However, the HADS is not a standardized criterion for the diagnosis of anxiety or depression, which may result in false positive results. Second, many demographic and clinical characteristics of the patients were included. Nevertheless, the sample size is relatively small, which may lead to bias regarding our results. In our future study, the relationship between PCI and anxiety and depression symptoms in CHD patients will be investigated by including a large sample of subjects. Third, follow-up assessments were performed in the outpatient department or *via* telephone. Most patients were followed up with in the outpatient department. However, a few patients were followed up with by telephone, for various reasons. Thus, we could not rule out that the follow-up interview through telephone may influence the results.

## Conclusions

Our current findings demonstrated that the symptoms of anxiety and depression change over time after PCI in CHD patients. Our results provide a framework for monitoring the symptoms of anxiety and depression *via* a non-pharmacologic intervention and a foundation for further investigations regarding the association between anxiety, depression and long-term prognosis after PCI. These findings are also conducive to achieving health objectives for PCI patients.

## Abbreviations

AMI, Acute myocardial infarction; CHD, coronary heart disease; HADS, Hospital Anxiety and Depression Scale; MACE, Major adverse cardiovascular events; PCI, percutaneous coronary intervention; TLR, Target lesion revascularization
